# An artificial intelligent single cell is part of the cell dream world

**DOI:** 10.1007/s10565-018-9433-1

**Published:** 2018-05-18

**Authors:** Yiming Zeng, Xiaoyang Chen, Hongzhi Gao, Xiangdong Wang

**Affiliations:** Department of Pulmonary and Critical Care Medicine, The Second Hospital of Fujian Medical University, Quanzhou, Fujian Province China

Cell biology and toxicology has in recent years received a tremendous growth of interest which has resulted in a disparity between the speeds that the technology and science has advanced in when comparing with its increased attention and importance. There are a multitude of factors that has led to this disparity such as a more complex cell structure that we initially imagined. Biological functions of a cell can be described in depth through integration of trans-omics with systems biology, on which the concept of clinical trans-omics was coined after fusing molecular multiomics, e.g., genomics, proteomics, and metabolomics, of cells/tissues harvested from patients with clinical phenomes (Wang X. 2018). With the development of the Human Cell Atlas project (Regev et al. 2017), the single-cell biology and systems biology will be painted out by measuring high-throughput single-cell molecular profiling. It is possible to identify single-cell characters involved with metabolic networks or signaling networks as well as complex interactions within the cell as an intact biological system. The early concept of single-cell biology was proposed for the discovery and development of disease-specific biomarkers (Niu et al. 2016), although we did not understand how to link single-cell measurements with clinical phenomes in order for patients’ to reap the benefits of molecular therapies. Niu et al. (2016) tried to tell a story of single-cell systems where molecular profiles (e.g., gene and protein expression and sequencing) as well as their networks and interactions could be well defined and organized into a larger picture.

Based on the knowledge of single-cell biology, computerized whole-cell models may be developed and established with the capacity for auto-learning for intelligent medicine and precision medicine. Furthermore, we call your special attention to think of the artificial intelligent (AI) single-cell as an optimal system with full understanding of cell molecular profiles, intelligent capacity for functioning and deep learning, and precise interpretation of measurements. Such an AI single-cell system could be expected to translate the message between single-cell molecular profiles and clinical phenotypes, explain alterations of single-cell gene/protein expression and networks in patient response to therapies, and act as a decision-making assistant for disease diagnosis and monitoring.

Artificial intelligent single cell is defined as a single-cell-like system with computerized databases, digitalized informatics of biological elements, and programmed function and signals. Koch recently addressed that artificial intelligence is coming to our life and should be considered to be a “natural” part of reality (2018). A machine-learning system based on AI which has entered the clinical practice, known as “Intelligent Medicine,” which aims to assist clinicians in the analysis of images (e.g., pathology, computed tomography, ultrasounds, echocardiograms) and clinical phenomics, and benefit patients through AI-based organ-like products. AI-based image readers can digitally re-image the small tumor within the lung according to the size, density, edge, clearness, and other digital information in order to make an early diagnosis of lung cancer. Furthermore, it seems possible that the AI single cell can also read and illustrate the messages of gene/protein expression or sequencing with a computer vision, and digest and analyze large amount of trans-omics data with the capacity of deep learning. With the significant improvement of single-cell isolation and purification, it is possible to build up a computerized database with genomic expression and sequencing, proteomic expression and activation, lipidomic profiles and metabolites, glycomic elements and function, and signal network and interaction, as mentioned previously (Niu et al. 2016).

AI single cell is increasingly imminent, since digitalized informatics of biological elements can be programmed and constructed. Mohammadi et al. (2018) developed an archetypal analysis for cell-type identification (ACTION) as an important and innovative approach for defining cell types, functional identity, and underlying regulatory factors from single-cell expression. This can be an example or part of AI single cell functioning, e.g., to discover cell identity and subtype on the basis of measured transcriptional profiles and their dominant functions, as well as reconstructed regulatory networks and interactions. The primary form of AI single cell should be more focused and simplified based on a certain function, e.g., one of the cell identity, subtype, mutation, or signal function, while remaining repeatable and standardized for clinical practice. For example, the ACTION system includes the biologically inspired metric, geometric approach, automated mechanism, orthogonalization procedure, and statistical analysis, to mainly identify cell subtypes (Mohammadi et al. 2018). AI single cells should be labeled and classified according to their function and be generated clinically as necessary.

AI single cell will become a clinical assistant decision-making system, which will aid in diagnosing and monitoring patients. The established AI single cell model is expected to describe or predict cell identity and dysfunction and propose strategies for precision medicine. AI single cell is used to detect cell biological behaviors or activities and assist in developing and validating disease-specific markers for diagnosis and treatment. AI single cell should have unique simulation engines, optimization operations, and interpretation of the characteristics of parameters, which can be collected and deep-learned from each measurement. Cadwell et al. (2017) developed a novel approach to collect a combination set of measures, e.g., whole-cell patch clamp recording for electrophysiological properties, immunohistochemistry for morphological phenotypes, and single-cell RNA sequencing for gene expression patterns. The outstanding points from this study is that they aim to integrate the function, morphology, and gene expression profile from single neurons and provide a new indication for AI single cell with multidimensional phenotypic variability. One of the most challenging obstacles to developing an AI single-cell system is how to better analyze complex functions, make experimental traceability, and monitor data quality and viability. It will be even more challenging to complete model construction, integration, and verification for visual analysis and applicable value of early detection and therapeutic evaluation and for integration of patient histopathology, clinical treatment, and imaging examination, in order to achieve the role of clinical assistant decision-making.

In conclusion, the artificial intelligent single cell is defined as a single-cell-like system with computerized databases, digitalized informatics of biological elements, and programmed function and signals. The artificial intelligent single cell can act as an optimal system with a full understanding of cell molecular profiles, intelligent capacity of functioning and deep learning, and precise interpretation of measurements. Such systems can translate the message between single-cell molecular profiles and clinical phenotypes, explain alterations of single-cell gene/protein expression and networks in patient response to therapies, and act as a decision-making assistant for disease diagnosis and monitoring.
